# The impact of frequent napping and nap practice on sleep-dependent memory in humans

**DOI:** 10.1038/s41598-018-33209-0

**Published:** 2018-10-10

**Authors:** Elizabeth A. McDevitt, Negin Sattari, Katherine A. Duggan, Nicola Cellini, Lauren N. Whitehurst, Chalani Perera, Nicholas Reihanabad, Samantha Granados, Lexus Hernandez, Sara C. Mednick

**Affiliations:** 1 Department of Psychology, University of California, Riverside, Riverside, CA 92521 USA; 2 Princeton Neuroscience Institute, Princeton University Princeton, NJ, 08544 USA; 3 Department of Cognitive Sciences, University of California, Irvine Irvine, CA, 92697 USA; 4 Department of Psychiatry, University of Pittsburgh School of Medicine Pittsburgh, PA, 15261 USA; 5 Department of General Psychology, University of Padova Via Venezia 8, Padova, CA, 315131 Italy

## Abstract

Napping benefits long-term memory formation and is a tool many individuals use to improve daytime functioning. Despite its potential advantages, approximately 47% of people in the United States eschew napping. The goal of this study was to determine whether people who endorse napping at least once a week (nap+) show differences in nap outcomes, including nap-dependent memory consolidation, compared with people who rarely or never nap (nap−). Additionally, we tested whether four weeks of nap practice or restriction would change sleep and performance profiles. Using a perceptual learning task, we found that napping enhanced performance to a greater degree in nap+ compared with nap− individuals (at baseline). Additionally, performance change was associated with different electrophysiological sleep features in each group. In the nap+ group, spindle density was positively correlated with performance improvement, an effect specific to spindles in the hemisphere contralateral to the trained visual field. In the nap− group, slow oscillatory power (0.5–1 Hz) was correlated with performance. Surprisingly, no changes to performance or brain activity during sleep emerged after four weeks of nap practice or restriction. These results suggest that individual differences may impact the potential benefits of napping on performance and the ability to become a better napper.

## Introduction

Sleep plays an important role in stabilizing or enhancing memory for newly learned information (i.e., memory consolidation)^1^. Daytime naps are sometimes as effective as nocturnal sleep in facilitating these memory processes^2–4^. Performance enhancement effects from naps have been found across a wide range of cognitive abilities, including episodic memory^5,6^, emotion regulation^7,8^, procedural skills^9^, and attention^10^. Napping has also been endorsed as a way to boost creativity^11,12^ and productivity^13^, improve performance in athletes^14^, and help people cope with fatigue-related to shiftwork^15–17^.

Despite the demonstrated benefits of napping, not everyone naps. In the United States, the National Sleep Foundation Sleep Health Index 2014^18^ reported that 53% of adults nap regularly, defined as having napped at least once in the past 7 days. Though there is no consensus for what defines a “napper” or “non-napper”, napping behavior in a young, healthy population likely follows different regulatory patterns and environmental opportunities than other populations that report frequent napping, e.g., infants, preschool children, older adults, and sleep-disordered individuals^19–24^. In young, healthy adults, consistent patterns in subjective reports and objective measures (e.g., sleep electroencephalography (EEG)) have emerged suggesting that there may in fact be differences between people who endorse napping and people who do not^25^. Non-nappers often report that they eschew the practice because they wake up feeling groggy, unproductive, and do not receive any benefits from a nap^26^. At the physiological level, this post-waking cognitive impairment (i.e., sleep inertia) may be associated with the amount of slow wave sleep (SWS) and waking from SWS^27^. Indeed, we previously found that non-nappers spend more time in deep SWS, whereas frequent napping was associated with more time in light, Stage 1 and Stage 2 sleep during a daytime nap^28^. Thus, day-to-day experiences with napping may change the quality of daytime sleep, which may, in turn, impact memory processing during the nap and symptoms of sleep inertia upon awakening.

Here, we investigated the impact of prior napping (nap+ vs. nap−) on nap-dependent memory consolidation and sleep inertia. Additionally, we used a cross-over design to test if four weeks of “nap practice” (at least 3 naps/week) or “nap restriction” in nap+ and nap− individuals would alter sleep physiology and performance profiles. Participants took an in-lab polysomnographically-recorded (PSG) nap at three different time points during the study – baseline, mid-intervention and post-intervention. On each in-lab nap day, pre- and post-nap behavioral performance was measured using a perceptual learning task (texture discrimination) that has shown the same magnitude of sleep-dependent memory benefit from a nap as a full night of sleep^2^. We also tested post-nap cognitive functioning using a descending subtraction test^29,30^, and collected ratings of subjective sleepiness throughout the day.

We first aimed to replicate the finding^2^ that same-day performance on a texture discrimination task only shows improvement following a nap by comparing individuals who napped (ignoring prior nap experience as in the original study) with individuals who remained awake. Critically, the nap group was comprised of both nap+ and nap− individuals. We hypothesized that by taking this difference in napping experience into account, we would see different performance outcomes between nap+ and nap− following a nap, including greater perceptual learning gains, less sleep inertia (as indexed by descending subtraction test performance) and less subjective sleepiness in nap+ individuals 4. To summarize, we hypothesized that nap+ participants would do better and feel better following a nap than nap− participants.

Our second aim was to examine how individual differences in sleep EEG features were associated with performance outcomes, namely minutes and percent of each sleep stage, sleep spindles, slow oscillation (0.5–1 Hz), delta (1–4 Hz) and sigma (12–15 Hz) power during non-rapid eye movement (NREM) sleep, and theta (4–8 Hz) power during rapid eye movement (REM) sleep. Prior work suggests that multiple consolidation processes, not necessarily mutually exclusive, may underlie perceptual learning. Each of these proposed processes tends to be linked to different sleep features, e.g., synaptic homeostasis and slow wave activity^31^, recovery from perceptual deterioration and SWS^2^, reactivation during NREM sleep^32^ and sleep spindle activity^33^ (i.e., active systems consolidation hypothesis)^1^, synaptic strengthening that leads to improvement above and beyond the performance level achieved during training, which may depend on a combination of NREM and REM sleep^2,34–37^. Given the overall complexity of this picture, we did not think that group level differences (e.g., increased SWS in nap−) would sufficiently explain why one group might receive more perceptual learning benefits from a nap. Rather, we hypothesized that prior nap experience would moderate the associations between sleep features and performance outcomes. Specifically, because we thought nap+ individuals would show more nap-related performance improvement, we expected that sleep EEG features linked to task improvement (spindles and REM sleep) would be more strongly associated with performance changes in the nap+ compared with the nap− group.

Finally, we aimed to test if nap-related learning benefits are experience-dependent. We hypothesized that napping is a trainable skill, such that nap− individuals who practiced napping for four weeks would show more perceptual learning gains, less sleep inertia and less subjective sleepiness following a daytime nap.

## Results

### Visit 1: Does prior napping affect post-nap performance, sleep brain activity, and sleep inertia?

#### Perceptual Learning: Nap vs. Wake

**Figure 1:**
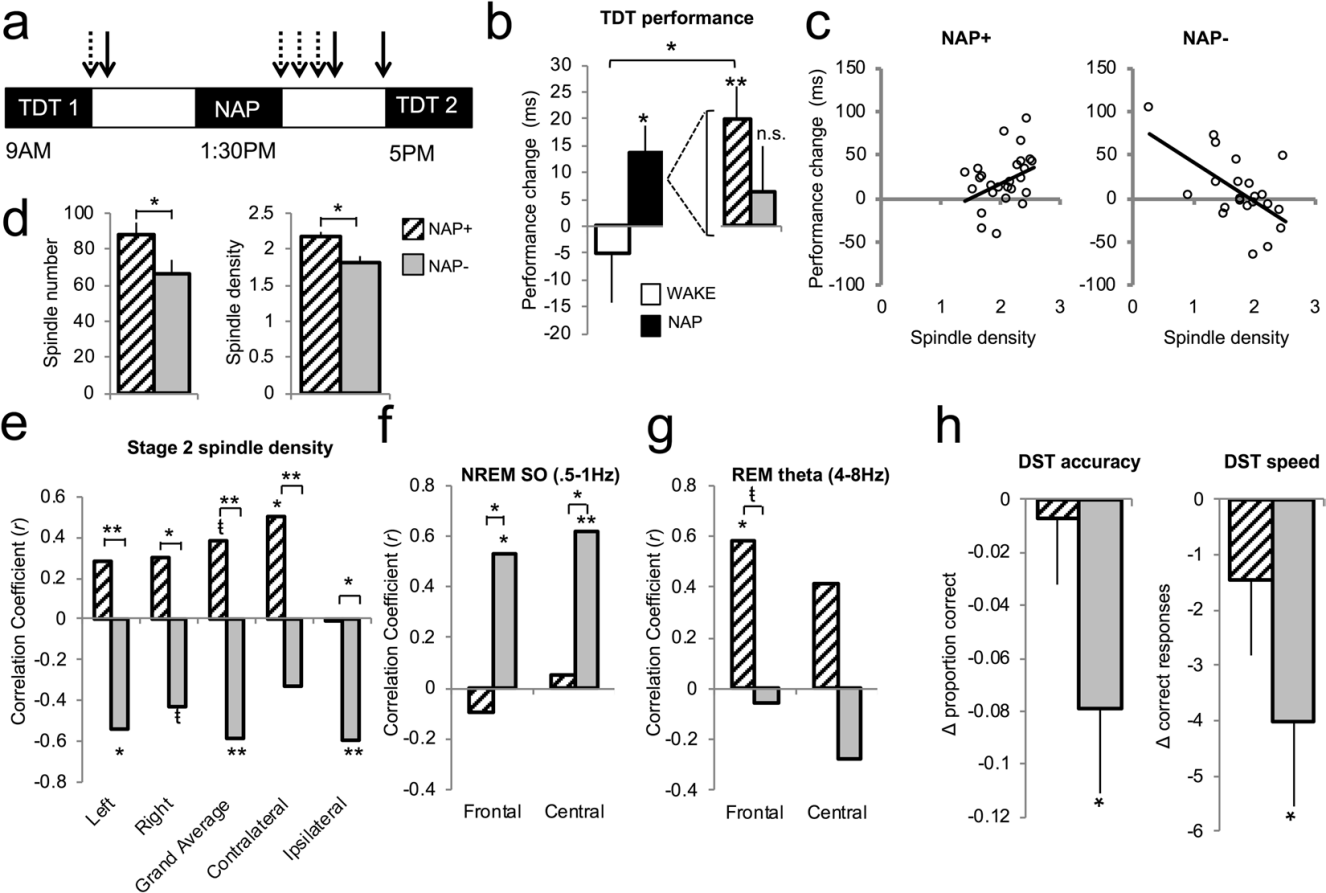
Visit 1 Baseline. (**a**) In-lab test day procedure. Texture discrimination task (TDT) thresholds were obtained at 9AM and 5PM. All participants napped between 1:30–3:30PM. Solid arrows indicate times of Karolinska Sleepiness Scale (KSS) administration; dashed arrows indicate times of the descending subtract test (DST) administration. (**b**) TDT threshold difference at baseline for the Wake (*n* = 21, white bar) and Nap (*n* = 48, black bar) groups. Within the Nap group, only nap+ (*n* = 26, hatched bar) showed learning; nap− (*n* = 22, solid gray bar) did not show significant improvement. All subsequent panels have the same number of independent data points (*n*) represented in each group unless noted. (**c**) Performance improvement was correlated with Stage 2 spindle density (grand average plotted), but in opposing directions for nap+ (*r* = 0.38, *p* = 0.055) and nap− (*r* = −0.59, *p* = 0.004). (**d**) Nap+ had more spindles (*p* = 0.04) and greater spindle density (*p* = 0.008) during Stage 2 than nap− (C3-P3_avg_ shown). (**e**) Correlation coefficients (Pearson *r*) for correlations between performance improvement and Stage 2 sleep spindle densities (note: In the nap− group, the Left correlation is *n* = 20 and Contralateral and Ipsilateral correlations are both *n* = 21, due to missingness from bad electrodes). There were also differing  associations between performance and (**f**) NREM slow oscillation power (SO, 0.5–1 Hz) and (**g**) REM theta power (4–8 Hz) based on nap+/nap− grouping. For panels (e), (f) and (g), an asterisk above or below the bar indicates a significant correlation; brackets indicate significant differences in *r*-values between groups (note: NREM SO correlation in nap− had *n* = 21; REM theta correlations had nap+ frontal *n* = 15, nap+ central *n* = 17, nap− frontal *n* = 16, nap− central *n* = 15; reduced *n* due to naps not containing REM sleep and/or bad electrodes). (**h**) DST performance change 5 min after awakening from the nap (note: nap+ *n* = 25 and nap− *n* = 20; reduced *n* due to experimenter error and performance that did not meet inclusion criteria). *ŧ indicates p* ≤ 0.07, * indicates p < 0.05, **indicates p < *0.005. Error bars are* ±*1 SEM*.

Participants completed a texture discrimination task (TDT) two times on the same day, once in the morning and once in the evening, while either remaining awake or taking a nap between task sessions. We computed the difference in thresholds between sessions, and replicated the classic finding^2^ that a nap enhances TDT performance compared to wake [*t*(67) = 1.95, *p* = 0.056, Cohen’s *d* = 0.49, independent samples *t*-test]. Discrimination thresholds significantly improved in participants who napped [*t*(47) = 2.69, *p* = 0.01, one-sample *t*-test] and were not different from zero in participants who remained awake [*t*(20) = −0.58, *p* = 0.57, one-sample *t*-test] (Fig. 1b). Change in performance was positively correlated with SWS min (*r* = 0.36, *p* = 0.01) and SWS% (*r* = 0.32, *p* = 0.03), as well as the product of SWS and REM min (SWSxREM, *r* = 0.34, *p* = 0.02). The latter result is consistent with the classic studies we aimed to replicate here^2,36^, although we note this may not be the best way to statistically test the combined contribution of SWS and REM to performance improvement^37^. Also, learning was negatively correlated with Stage 2% (*r* = −0.33, *p* = 0.02), which may reflect a trade-off between Stage 2 and SWS (amounts of Stage 2 and SWS were inversely correlated; minutes: *r* = −0.45, *p* = 0.001, percent: *r* = −0.69, *p* < 0.001).

#### Perceptual Learning: Nap+ vs. Nap−

We re-analyzed the above data, now separating the nap group into two subgroups based on prior nap experience (nap+ and nap−). As predicted, nap+ showed significant performance gains after a nap [*t*(25) = 3.38, *p* = 0.002, one-sample *t*-test], whereas performance change in nap− did not differ from zero [*t*(21) = 0.74, *p* = 0.47, one-sample *t*-test] (Fig. 1b), although the difference between the two groups did not reach statistical significance [*t*(46) = 1.35, *p* = 0.18, Cohen’s *d* = 0.39, independent samples *t*-test). Compared with the wake group, only the nap+ group was significantly better [nap+ vs. wake: *t*(45) = 2.42, *p* = 0.02, Cohen’s *d* = 0.70, independent samples *t*-test; nap− vs. wake: *t*(41) = 0.93, *p* = 0.36, Cohen’s *d* = 0.28, independent samples t-test]. These effects were not due to differences in pre-nap thresholds between wake, nap+ and nap− groups [*F*(2,66) = 0.79, *p* = 0.46, one-way ANOVA]. Together with the above results, this analysis demonstrates that the nap-dependent improvement observed in the Nap vs. Wake analysis was driven by nap+ individuals, and suggests that not all individuals show learning benefits from daytime sleep.

#### Daytime Sleep Architecture

During the first in-lab visit (prior to the intervention group assignment), we found no significant differences in total sleep time or minutes or percent of any sleep stage between nap+ and nap− (all *p*s > 0.29) (Supplementary Table 2). Since daytime sleep may be directly related to nighttime sleep, we also compared the prior night’s sleep between nap+ and nap−, using actigraphy, and found no differences (Supplementary Table 3). Additionally, total sleep time the night before the experimental day did not correlate with nap sleep stages in either group (all *p*s > 0.17), or the sample as a whole (all *p*s > 0.14).

Differences were noted, however, in sleep spindle events during the nap. Nap+ had approximately 31% more sleep spindle events during Stage 2 sleep compared to nap− [C3-P3_avg_, nap+ mean 87.6 (SD = 34.5) vs. nap− mean 66.8 (SD = 33.7) spindle events, *t*(46) = 2.11, *p* = 0.04, Cohen’s *d* = 0.61, independent samples *t*-test] (Fig. 1d). Given inter-subject variability in duration of Stage 2 and SWS, we focused our remaining analyses on spindle density (spindle count/minutes). In the left hemisphere, nap+ showed greater spindle density than nap− for Stage 2 sleep [C3-P3_avg_, nap+ mean 2.2 (SD = 0.4) vs. nap− mean 1.8 (SD = 0.5) spindles/min, *t*(46) = 2.79, *p* = 0.008, Cohen’s *d* = 0.80, independent samples *t*-test], but no differences in the right hemisphere (C4-P4_avg_, *p* = 0.41). Also, no differences in sleep spindles were identified during SWS in either hemisphere (all *p*s > 0.10).

Next, we examined spectral power in specific frequency bands of interest (NREM SO, delta, and sigma; REM theta) over frontal (F3-F4_avg_) and central (C3-C4_avg_) electrode sites. There were no differences between nap+ and nap− in any frequency band during NREM sleep, however, for REM sleep, nap+ had greater theta power [*t*(30) = 2.15, *p* = 0.04, Cohen’s *d* = 0.77, independent samples *t*-test) over central sites. Together, these results indicate that nap sleep physiology was equivalent in nap+ and nap− groups across most sleep variables, with the exception of left hemisphere spindles and REM theta power. In the following section, we take a closer look at how the relation between sleep features and behavior might differ in these groups.

#### Brain-Behavior Relationships: Nap+ vs. Nap−

We next examined associations between sleep features and performance separately for nap+/− participants, and tested for a moderating effect of nap frequency group on these associations. Prior nap experience did not moderate the association between sleep stages and performance change, with both nap+ and nap− showing similar direction and magnitude of correlation coefficients. However, there were substantial differences between the groups in how sleep spindles were associated with performance (Fig. 1c,e). In nap−, performance was consistently negatively correlated with spindle density (C3-C4-P3-P4_avg_) during Stage 2 (*r* = −0.59, *p* = 0.004), SWS (*r* = −0.37, *p* = 0.13, negative correlation, but non-significant), and NREM stages combined (*r* = −0.62, *p* = 0.002). In nap+, there was a trending positive performance-spindle density relationship for Stage 2 (*r* = 0.38, *p* = 0.055), and no significant relationship for SWS or NREM combined spindles (SWS: *r* = −0.11, NREM: *r* = 0.16; both *p*s > 0.45). The statistical difference between correlation coefficients in the two groups was significant for Stage 2 sleep spindles (*z* = 3.47, *p* = 0.0005) and NREM combined (*z* = 2.84, *p* = 0.004), indicating that nap group moderated the relation between spindles and performance change.

Given that learning on the TDT is retinotopically-specific^38^, we further examined Stage 2 spindles in the ipsilateral and contralateral hemispheres relative to the trained visual field location. Nap+ showed a pattern of results predicted by a retinotopic learning effect, with a positive correlation between performance and contralateral spindles (*r* = 0.50, *p* = 0.009) and no significant relation between performance and ipsilateral spindles (*r* = −0.008, *p* = 0.97). In nap−, both contralateral (*r* = −0.33, *p* = 0.14) and ipsilateral (*r* = −0.60, *p* = 0.004) spindles were negatively correlated with performance, although this association was only significant for ipsilateral spindles. There was significant moderation for both the contralateral (*z* = 2.84, *p* = 0.004) and ipsilateral (*z* = 2.16, *p* = 0.03) effects.

Interestingly, NREM SO and delta power strongly correlated in the positive direction with performance change in nap− (SO frontal: *r* = 0.53, *p* = 0.013; SO central: *r* = 0.62, *p* = 0.003; delta frontal: *r* = 0.52, *p* = 0.015; delta central: *r* = 0.60, *p* = 0.005), but not in nap+ (SO frontal: *r* = −0.10, *p* = 0.63; SO central: *r* = 0.06, *p* = 0.79; delta frontal: *r* = 0.01, *p* = 0.95; delta central: *r* = 0.21, *p* = 0.30) (Fig. 1f). We note that the strong correlations within the nap− group were potentially driven by SO and delta power during SWS, which consistently showed larger magnitude correlation coefficients relative to Stage 2 (SO frontal: Stage 2 *r* = 0.02 vs. SWS *r* = 0.38; SO central: Stage 2 *r* = 0.12 vs. SWS *r* = 0.45; delta frontal: Stage 2 *r* = −0.05 vs. SWS *r* = 0.22; delta central: Stage 2 *r* = 0.11 vs. SWS *r* = 0.47). Correlation coefficients significantly differed between groups for NREM SO power over frontal (*z* = 2.20, *p* = 0.03) and central (*z* = 2.11, *p* = 0.03) sites; NREM delta power correlations did not significantly differ (frontal: *p* = 0.07, central: *p* = 0.10). NREM sigma power negatively correlated with performance only in nap− (frontal: *r* = −0.45, *p* = 0.03; central: *r* = −0.38, *p* = 0.08, non-significant), not in nap+ (frontal: *r* = −0.01, *p* = 0.96; central: *r* = 0.15, *p* = 0.46). However, tests for moderation were non-significant (frontal: *p* = 0.10; central: *p* = 0.08). REM theta power was positively correlated with performance in nap+ (frontal: *r* = 0.58, *p* = 0.02; central: *r* = 0.41, *p* = 0.10), but not in nap− (frontal: *r* = −0.05, *p* = 0.84; central: *r* = −0.28, *p* = 0.32) (Fig. 1g), with non-significant tests for moderation (both *p* = 0.07). Unlike the spindle result, we did not find retinotopically-specific differences in any of the frequency bands, which may be related to the more localized nature of spindles compared to more global oscillations that occur during sleep^39–42^. In summary, these results reveal differences in the underlying oscillatory circuitry associated with consolidation mechanisms during daytime sleep for nap+ and nap−. Specifically, we found that prior nap frequency significantly moderated the brain-behavior relationship between contralateral Stage 2 spindles and learning, and NREM SO power and learning.

#### Subjective sleepiness and sleep inertia: Nap+ vs. Nap−

Since non-nappers often report not enjoying napping^26^, and anecdotally complain of feeling groggy and unproductive after a nap, we measured subjective sleepiness and cognitive functioning after the nap. Participants rated their subjective sleepiness at three time points across the study day: 1) pre-nap, 2) 10 min post-nap, and 3) 90 min post-nap (see Fig. 1a). Overall, subjective sleepiness decreased across the day [*F*(2,82) = 16.46, *p* < 0.001, partial eta^2^ = 0.29, mixed ANOVA main effect], with a slight reduction in sleepiness, or boost in alertness, evident 10 min post-nap compared to pre-nap (trending at *p* = 0.06, paired *t*-test), and an even further reduction in sleepiness approximately 90 min after waking compared to 10 min post-nap (*t*(44) = 4.81, *p* < 0.001, paired *t*-test). There was no main effect of group (*p* = 0.36, mixed ANOVA) and no timepoint x group interaction (*p* = 0.88, mixed ANOVA), indicating that both groups received similar boosts in alertness from the nap.

We probed the degree of sleep inertia experienced by participants using a descending subtraction test (DST)^29,30^ to measure cognitive functioning at 11AM and again 5, 20 and 35 min after awakening from the nap (see Fig. 1a). At 11AM (prior to the nap), there were no differences in speed (total number of correct responses) or accuracy (correct responses/total responses) between nap+/− (both *p*s > 0.55, independent samples *t*-test). The task proved to be sensitive to impairment in cognitive functioning upon awakening, with differences in speed across the day [*F*(3,120) = 14.08, *p* < 0.001, partial eta^2^ = 0.26, mixed ANOVA main effect], including a significant dip in performance 5 min post-nap compared to pre-nap (post hoc *p* = 0.005) and recovered performance by 20 min post-nap. Although accuracy showed the same general trend, it did not reach statistical significance [*F*(3,120) = 2.45, *p* = 0.07, partial eta^2^ = 0.06, mixed ANOVA main effect]. Neither speed nor accuracy showed a main effect of group (*p*s > 0.66) or timepoint × group interaction (*p*s > 0.44). Numerically, as predicted, the nap− group showed greater decrements than the nap+ group. We conducted further exploratory analyses within each group at each timepoint. Upon awakening from the nap, nap− showed significant decrements in speed [*t*(19) = 2.62, *p* = 0.02, paired *t*-test] and accuracy [*t*(19) = 2.44, *p* = 0.02, paired *t*-test] compared to their pre-nap performance (Fig. 1h). On the other hand, nap+ did not show significant impairment in speed [*t*(24) = 1.06, *p* = 0.30, paired *t*-test] or accuracy [*t*(24) = 0.28, *p* = 0.78, paired *t*-test] . By 20 min post-nap, DST speed increased in both groups relative to the 5-min assessment [nap−: *t*(20) = 3.43, *p* = 0.003; nap+: *t*(24) = 5.07, *p* < 0.001; paired *t*-tests]. Compared to pre-nap speed, nap+ were significantly faster (*t*(24) = 2.68, *p* = 0.01, paired *t*-test); on the other hand, nap− recovered, but did not improve, their speed (*t*(19) = 1.06, *p* = 0.30). By 35 min post-nap, the nap+ group maintained their improved speed relative to pre-nap [*t*(22) = 2.04, *p* = 0.05, paired *t*-test]; the nap− group showed a non-significant improvement in speed (*t*(18) = 1.91, *p* = 0.07, paired *t*-test). Accuracy did not show any significant differences at 20 min or 35 min post-nap compared to pre-nap in either group (all *p*s > 0.11). Overall these results suggest no sign of significant sleep inertia in nap+ participants, based on both subjective reports (KSS ratings) and objective performance (DST) measures. On the other hand, nap− participants, notwithstanding a similar perceived level of sleepiness as nap+, showed impaired cognitive functioning on the DST task, suggesting nap− individuals experience more performance-related sleep inertia upon awakening from a nap. However, it should be noted that these were post-hoc exploratory analyses following a non-significant omnibus test, and these results should be interpreted with caution.

### Does nap Practice/Restriction change performance profiles associated with napping?

Next, we investigated the effect of four weeks of nap Practice or Restriction. Participants in the Practice condition took 3.13 (SD = 0.90) naps per week on average. Weekly nighttime sleep descriptive statistics (from actigraphy) are provided in Supplementary Table 4. Statistics reported below are from 3 (visits 1/2/3) × 2 (nap+/nap−) × 2 (practice/restriction) mixed-model ANOVAs.

#### Behavioral performance

**Figure 2:**
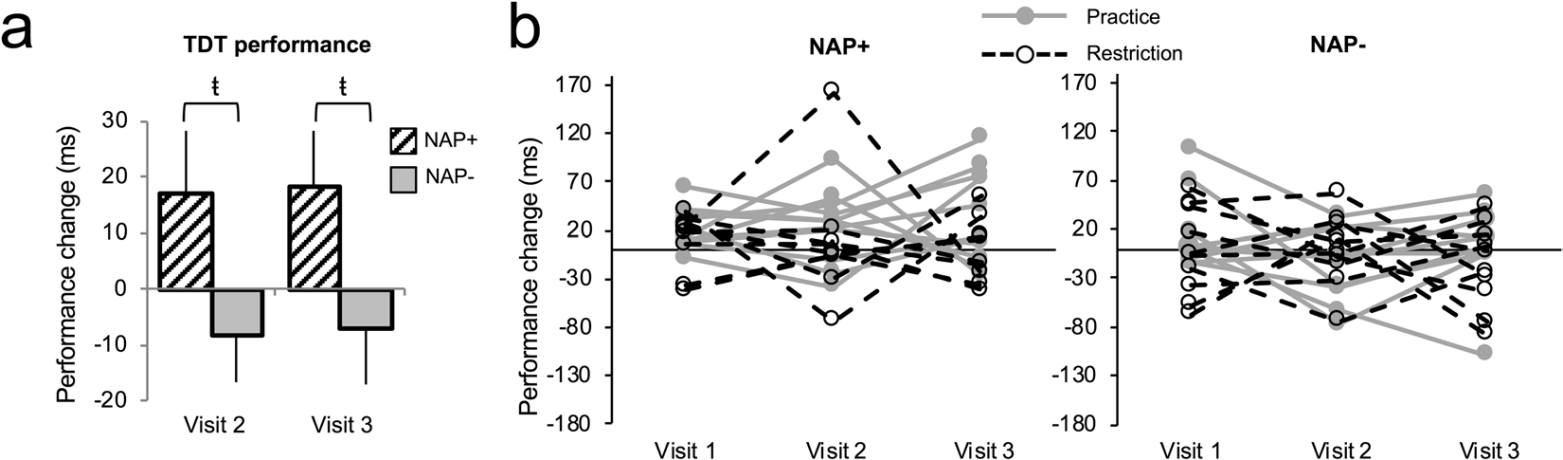
Nap practice/restriction intervention. (**a**) TDT threshold difference at Visit 2 and Visit 3. Nap+ (*n* = 20) always showed improvement and nap− (*n* = 20) did not. (**b**) Individual participant performance on the TDT is plotted across visits within nap frequency groups. It is visually apparent that the magnitude of nap-dependent memory improvement remains stable across the four-week nap Practice (gray lines) or nap Restriction (dotted black lines) intervention in both nap+ and nap− groups (nap+ Practice *n* = 11, nap+ Restriction *n* = 9, nap− Practice *n* = 10, nap− Restriction *n* = 10). *ŧ indicates p* ≤ *0.07*. *Error bars are* ±*1 SEM*.

For perceptual learning, in contrast with our prediction, there was no main effect of visit or intervention (practice/restriction) and no significant interactions, indicating that neither nap practice nor restriction altered performance across time. There was, however, a main effect of nap frequency group [*F*(1,36) = 5.61, *p* = 0.02, partial eta^2^ = 0.14, mixed ANOVA], which revealed that nap+ always showed more performance gains with a nap than nap− collapsing across visits [although note that, similar to Visit 1, the difference between groups at each visit did not reach traditional statistical significance levels, but showed a medium strength effect size [Visit 2: *t*(38) = 1.83, *p* = 0.074, Cohen’s *d* = 0.58, independent samples *t*-test; Visit 3: *t*(38) = 1.83, *p* = 0.075, Cohen’s *d* = 0.58, independent samples *t*-test] (Fig. 2a). In other words, the differential performance outcomes observed at Visit 1 (baseline) were maintained throughout the study (Fig. 2b). On each visit, pre-nap thresholds were comparable between nap+ and nap− (all *p*s > 0.20).

#### Daytime sleep architecture

We found no differences in nap total sleep time (*p* = 0.88), Stage 1 (*p* = 0.49), Stage 2 (*p* = 0.88), SWS (*p* = 0.57), or REM (*p* = 0.67) (reported *p*-values are from the 3 × 2 × 2 interaction) minutes across the intervention (Supplementary Table 5). Likewise, there were no changes in spindle densities (Stage 2 C3-C4-P3-P4_avg,_ *p* = 0.30) or power spectra (NREM SO: *p* = 0.64, NREM delta: *p* = 0.76, REM theta: *p* = 0.89) as a function of nap practice/restriction. These results indicate that short-term changes to nap frequency did not have a significant impact on the architecture of daytime sleep.

#### Subjective sleepiness and sleep inertia

Across the four weeks, there were no changes in subjective sleepiness after waking from the nap as a function of nap practice or restriction (*p* = 0.56). The degree of post-nap sleep inertia as measured by the DST remained stable across visits, with no changes due to nap practice or restriction (Δspeed: *p* = 0.57; Δaccuracy: *p* = 0.19).

## Discussion

This young, healthy sample of self-reported nappers (nap+) demonstrated perceptual learning with a nap, whereas self-reported non-nappers (nap−) showed no performance improvement with a nap. Importantly, when these two subgroups were combined in the same analysis (Nap vs. Wake), it appeared as though, compared with a wake control group, a nap benefited perceptual learning on this task, replicating prior work^2,43^. However, further analysis revealed that this result was driven by the nap+ individuals, masking the fact that not all naps are equal. Further, performance in the two groups was associated with different oscillatory features during sleep, exhibiting contrasting associations between behavioral performance and spindle density, as well as behavioral performance and SO power. Finally, four weeks of experimental nap restriction or practice was not sufficient to alter the performance profiles of either group, such that neither restricting naps in nap+ individuals decreased the cognitive benefits they derived from naps, nor did increasing naps in nap-participants boost nap-related perceptual learning. These findings suggest that nap preferences either require a longer period of training to produce measurable enhancement, or that nap preferences are not experience-dependent. These results may have far-reaching implications for a wide range of people, including sleep researchers, as well as administrators, educators, policy makers, and clinicians who may recommend occupational napping.

Although there is no agreed upon definition for habitual napping, prior studies have found that the sleep architecture in people who nap more frequently is predominated by light sleep stages, whereas infrequent napping is associated with naps containing more SWS^25,44^. We previously reported a dose-dependent effect of napping on nap sleep architecture—more napping during a one-week period was associated with greater amounts of Stage 1 and 2, and less SWS^28^. In the current study, we did not find macroarchitecture sleep stage differences, perhaps due to a smaller range of prior nap frequency in this sample compared to McDevitt *et al*.^28^. However, we did find that nap+ participants had increased Stage 2 sleep spindles, and spindle density was associated with better performance in the nap+ group only. This correlation was especially strong for spindles occurring in the hemisphere contralateral to the trained target location. While this result may potentially reflect spindle activity localized to the brain area involved in learning^33,45^, it is important to consider the limited spatial resolution of scalp EEG and the limited number of electrodes used in this study. Nonetheless, this is a promising result that suggests future research should pursue investigating the role of sleep spindles for visual perceptual learning.

What might be the role of spindles for this type of learning? Spindle activity has been correlated with better performance on a wide range of memory tasks^46,47^, including studies in which prior nap preference is unspecified^4,48–51^ or primarily tested regular nappers^24,44^. Spindles are thought to reflect replay or reactivation of newly learned information, leading to transfer of information between brain networks and strengthening or modification of synaptic connections (active systems consolidation model)^46^. In the perceptual learning domain, increased power in the sigma band (i.e., spindle frequency) has previously been associated with performance improvement in humans^33^. Following perceptual learning in rodents, spindle oscillations were critical for relaying information between the thalamus (lateral geniculate nucleus) and primary visual cortex, ultimately leading to sleep-dependent response changes in V1^52^. Thus, spindles may be an electrophysiological marker of a consolidation process that reactivates information and strengthens synaptic connections in the perceptual learning network, leading to improved behavioral performance. It is possible that this reweighting could be occurring in low-level visual areas, at higher-level decision-making units, or both^53–55^.

The positive spindle correlation in nap+ individuals and significant moderation was specific to Stage 2 sleep. Studies tend to differ in whether they report memory associations with Stage 2 spindles^48,49,56,57^, SWS spindles^51^, or spindles in NREM sleep combined^4,50,58,59^. Prior work in the perceptual learning domain found that spindle activity during Stage 2 sleep, but not SWS, played a critical role^33^. Why might Stage 2 spindles, rather than SWS spindles, be more strongly related to the sleep-based consolidation examined in this study? A model by Genzel and colleagues^39^ proposes that light NREM sleep (i.e., Stage 2) may favor global information exchange and active potentiation, whereas SWS promotes local weakening of synaptic units (synaptic homeostasis, to be discussed further below). In their model, spindles do not drive the global replay event (cf.^60^). Rather, spindles immediately follow these replay events and trigger local synaptic plasticity in cortical networks involved in the preceding replay event. Extending their model to the current results, we posit that Stage 2 may favor consolidation of visual perceptual learning by facilitating global thalamocortical communication (e.g., replay between thalamus and visual cortex)^52^ and plasticity in cortical synapses (e.g., long-term potentiation in visual cortex)^61^.

It is also likely that there are other consolidation mechanisms at work during sleep^39^. For example, prior studies have shown that repeated training on the texture discrimination task without sleep typically leads to perceptual deterioration; and while a nap with NREM sleep can reverse the deterioration, REM sleep is required for performance improvement above baseline^43,62–65^. Notably, in the current study, post-nap performance in nap− participants did not significantly deteriorate between sessions, and nap− performance was correlated with power in slow frequency bands, which has been demonstrated to be critical for perceptual learning^31^. This suggests that even if nap− individuals did not have spindle-related performance gains, they may have still benefitted from slow wave-related perceptual maintenance. One potential mechanism comes from the synaptic homeostasis hypothesis (SHY), which posits that low frequency slow wave activity during sleep downscales synaptic connections that have become potentiated during wake^66,67^. In terms of learning, SHY postulates that encoding during wake increases potentiation of synapses in associated cortical networks, and during subsequent sleep, slow waves “downscale” the synaptic weight, with weaker synapses receiving relatively more downscaling than strong synapses. This process is proposed to increase the signal-to-noise ratio between strong and weak synapses, and, consequently, improve memory processing^68^. It is possible that slow waves may be important for reducing perceptual deterioration through a similar downscaling mechanism^31^.

Prior work has shown that REM sleep is critical for performance improvement on a texture discrimination task^2,34^. In the current study, only nap+ showed learning after the nap. Although the majority of both nap+ (*n* = 20) and nap− participants (*n* = 18) had REM sleep, and equivalent amounts of REM sleep at the group level, REM theta activity indicated that perhaps there was a qualitative difference in REM sleep between the groups. Specifically, REM theta power was increased in nap+, and correlated with performance improvement in nap+ only. Theta oscillations are known to be involved in memory processing during wakefulness^69^, and there is now increasing evidence to suggest it is involved in memory reprocessing during sleep. EEG theta activity was increased following learning of word pairs^70^ and emotional pictures^71^. Other work has shown that theta was increased after presenting memory reactivation cues during sleep^72,73^, and that REM frontal and temporal theta had high discriminative power for decoding what type of information people had learned prior to sleep^74^. Together, the spindle and theta results in nap+ participants suggest that napping may increase the potential for sleep to engage neural mechanisms related to memory reprocessing in these individuals. Furthermore, these data suggest that consolidation processes during NREM sleep (e.g., spindles) and REM sleep (e.g., theta) both contribute to perceptual learning and expand upon the two-process model proposed by Stickgold and colleagues^36^.

Contrary to our hypothesis, the four-week nap practice intervention produced no changes across any of the four outcomes of interest: nap sleep architecture, behavioral performance, sleep inertia, and subjective sleepiness. It is possible that our intervention was not long enough or did not require enough practice (minimum 20 minutes, 3 times per week for four weeks). Indeed, there is considerable variation in how long it takes people to form a habit^75^, with one study showing an average of 66 days (range 18 to 254 days) to form either an eating, drinking or activity behavior, and the length of our intervention was significantly shorter than this. However, it should be noted that habit formation studies measure automaticity of a response given a cue in the environment, which may be different from measuring cognitive benefits from frequent napping. In the current study, we did not collect self-reports that would allow us to examine automaticity of the behavior (e.g., if people came to “look forward to” or “rely” on their nap) in a habit formation framework. However, in regards to the four outcomes, there was no hint of a trend toward a change in performance for nap-individuals assigned to the practice group, suggesting that they were not on a trajectory toward change should the intervention have been extended. One potential caveat is that we were not able to obtain a reliable estimate of nighttime sleep across the intervention. We feel this is due to two factors. First, we asked people to follow their regular sleep schedules (within a two-hour bedtime window and a two-hour wake time window); participants were made aware that if they deviated from this schedule they would become ineligible to continue in the study. Thus, our participants were much more mindful of their nighttime sleep schedule and not entirely subject to “free-living” conditions. This may have restricted our ability to detect variations in overnight sleep characteristics due to nap practice or restriction. Second, the actigraph devices frequently malfunctioned, resulting in substantial missingness and not enough data in each cell (see Supplementary Table 4) to run inferential statistics. Therefore, our interpretation of how nap practice or restriction may have affected nighttime sleep is limited to patterns seen in the descriptive statistics and should be approached with caution. Nonetheless, an intriguing pattern that emerges from these data is that participants in intervention groups incongruent with their pre-existing nap preference (e.g., nap+ individuals in the nap restriction condition) showed changes in their nighttime sleep duration across the study. Nap+ individuals who were restricted from napping had an increase of ~17 minutes in overnight average total sleep time, whereas nap− individuals who practiced napping had a decrease of ~16 minutes. However, a similar decrease was seen in nap− individuals who were nap restricted, i.e., congruent with their pre-existing nap preference; nap+ individuals who continued to practice napping only increased ~2 minutes. This suggests that nighttime sleep may be altered to compensate for daytime sleep. However, a study specifically designed to answer this question would be more informative than the current study.

What might be some possible explanations for this individual difference in nap preference? Genetics are likely to play role, and one candidate is the clock gene *PERIOD3*, which contains a variable number tandem repeat polymorphism. This polymorphism has been linked to morningness/eveningness preference, delayed sleep phase syndrome, slow wave activity, and waking performance in response to sleep loss^76^. Napping is also related to these factors, and we speculate that *PERIOD3* may be one marker of people’s napping phenotype. Another possibility is that nap habits that arise early in development may have an effect on adult habits^23^. A recent study reported that napping was important for learning in preschool children, such that no learning occurred in children restricted from napping^24^. Closer inspection of the data revealed that the decreases in performance were only evident in habitual nappers restricted from napping, whereas no performance decrements were found in non-nappers. A working hypothesis that emerged from this study suggests that habitual nappers have an increased need for frequent consolidation, which may be related to brain maturation during development. Although it is not known how preschool nap habits may be related to adult nap habits, we can extend their hypothesis and posit that there may be functional differences in learning strategies in adults that place differential demands on cognitive load and increase the need for sleep throughout the day. Longitudinal studies that track nap patterns across the lifespan would be informative for understanding how nap habits develop and change (or do not change) over time. Another related question that arises is whether or not these differences in sleep-dependent consolidation extend to nighttime sleep. One possibility is that due to different factors regulating sleep (as discussed above), consolidation mechanisms (e.g., thalamocortical synchrony^46^ or spindle refractoriness^77^) are not optimized during daytime sleep in nap− individuals and consolidation is best accomplished overnight. Finally, further research is needed to determine how the present results may generalize to other populations, such as older adults and clinical samples, and how napping might impact other outcomes related to health and well-being^78^.

## Methods

### Participants

Eighty-three (51 F) healthy, non-smoking adults between the ages of 18 and 35 with no personal history of sleep disorders, neurological, psychological, or other chronic illness gave informed consent to participate in the study. All experimental procedures were approved by the Human Research Review Board at the University of California, Riverside. Methods were carried out in accordance with all guidelines and regulations.

Fifty-eight people participated in the experimental nap protocol explained below, and the twenty-five remaining participants were part of a one-day Wake control group. The Epworth Sleepiness Scale (ESS)^79^ and the reduced Morningness-Eveningness Questionnaire (rMEQ)^80^ were used to exclude potential participants with excessive daytime sleepiness (ESS score > 10) or extreme chronotypes (rMEQ < 8 or >21). All participants reported regularly going to bed no later than 2AM, waking up no later than 10AM, and getting at least 7 hours of total sleep per night on average. Heavy caffeine users (>3 servings per day) were not enrolled to exclude the possibility of significant withdrawal symptoms during the experiment. Nonetheless, one participant reported experiencing caffeine withdrawal symptoms and was excluded from analyses.

### General procedure

This was a five-week protocol that included one week of at-home baseline monitoring and four experimental weeks. Participants completed three in-lab study days, one each at the beginning (Visit 1), middle (Visit 2) and end (Visit 3) of the experimental period, spaced two weeks (14+/−2 days) apart. The study proceeded as follows: (i) Baseline week, (ii) in-lab Visit 1 at the end of the baseline week, with (iii) experimental group assignment (nap Practice or Restriction) occurring at the end of Visit 1 (see Nap Practice and Restriction section below), (iv) two experimental weeks following nap Practice/Restriction, (v) in-lab Visit 2, (vi) two experimental weeks following nap Practice/Restriction, and (vii) in-lab Visit 3. Participants in the Wake group only completed Visit 1.

During the study, participants agreed to maintain their habitual sleep-wake schedule (see above). Adherence to the sleep schedule was tracked with daily online sleep diaries and actigraph wrist monitors (Actiwatch Spectrum, Respironics) for the duration of the study, including the baseline week. Participants were asked to refrain from consuming caffeine, alcohol, and all stimulants for 24 hours prior to and including the study day.

### Study Day Procedure

On each in-lab study day, participants reported to the Sleep and Cognition Lab at the University of California, Riverside at 9AM. After verifying adherence to the sleep schedule by checking actigraphy data, participants completed Session 1 of a texture discrimination task (TDT).

At 12:30PM, electrodes were attached for standard polysomnographic (PSG) recording of sleep. All participants were given a two-hour nap opportunity between 1:30PM and 3:30PM to obtain up to 90 min of total sleep time. After reaching 90 min of total sleep time or after the two-hour nap opportunity time had elapsed, regardless of the current sleep stage, an experimenter ended the nap by knocking on the door and entering the bedroom (“nap end”). Lights remained off in the bedroom, and the participant was asked to continue lying supine for five minutes without falling back asleep while post-nap EEG and ECG measurements were made. If a participant spent more than 30 consecutive minutes awake during the nap window then the participant was removed from the bedroom and the nap was terminated.

The descending subtraction test (DST)^29,30^ was used to probe cognitive functioning due to sleep inertia. The task was administered at four time points, once before the nap (~11AM), and three times after the nap, specifically 5 min, 20 min, and 35 min after “nap end.” The 5 min DST time point was administered in the dark bedroom while the participant was lying supine; after this assessment, lights were turned on and the participant was free to sit up and move around the bedroom. The aim of this procedure was to obtain an initial assessment of post-nap sleep inertia (5 min time point) followed by two subsequent assessments (20 min and 35 min time points) to measure the dissipation of sleep inertia as the participant returned to normal daytime conditions.

At 5PM (Session 2), participants were re-tested on the TDT. Participants also completed the Karolinska Sleepiness Scale (KSS)^81^ at three times during the study day – (i) at the end of Session 1 (~11AM), (ii) 10 min post-nap (~3:40PM), and (iii) at the beginning of Session 2 (~5PM). Between sessions (~11AM to 12:30PM and ~4:10PM to 5PM for nap participants, and ~11AM to 5PM for Wake participants), participants left the lab and carried out their day as they normally would, but were instructed to not nap (verified through actigraphy), exercise, or consume caffeine or alcohol. Participants in the Wake group did not nap or complete the DST task or the 10 min post-nap KSS.

### Nap Frequency Groups

Due to lack of agreement across studies on how to categorize nappers from non-nappers, we opted to base our distinctions on self-assessment (based on questionnaire answers) and immediate prior evidence of napping (one week of sleep diaries and actigraphy). We obtained information about nap habits in multiple ways. First, during either a telephone or online survey screening questionnaire prior to study enrollment, participants were asked, “Do you take naps during the day? And if so, how many times per week? And how long do you nap?” Second, we counted the number of naps reported in participants’ sleep diaries during the baseline week prior to starting the study, and then verified that these naps occurred by checking the actigraphy data. We defined nap+ as people who reported napping at least once per week [mean 1.54 (SD = 1.03) naps per week], and nap− as napping less than once per week [mean 0.18 (SD = 0.40) naps per week; i.e., never napping or only napping once or twice a month^28,44^. When the two sources of information did not match (e.g., a participant reported never napping on the screening survey but then took a nap during the week prior to the study), the participant was interviewed about their nap habits by an experimenter who then made the determination. For example, if the participant reported being a non-napper but they napped because of illness that week, the participant retained nap− status since illness was an out of the ordinary event.

### Nap Practice and Restriction

Within each of these nap frequency groups, nap+ and nap− participants were randomly assigned to either a nap Practice or nap Restriction condition. Participants in the Practice group were instructed to nap at least three times per week for a minimum of 20 min for the remaining four weeks of the study (naps in the laboratory on study visits 2 and 3 were allowed to count toward their weekly nap total), whereas those in the Restriction group were instructed to not nap unless asked to take one in the lab during a study visit. Compliance to these conditions was verified by checking sleep diaries and actigraphy. One nap+ participant in the Restriction group took one nap during Week 2 due to illness; this participant’s data were retained in analyses.

### Polysomnography (PSG)

PSG data were collected using Astro-Med Grass Heritage Model 15 amplifiers and Grass Gamma software. Eight scalp electroencephalogram (EEG) and two electrooculogram (EOG) electrodes were referenced to unlinked contralateral mastoids (F3/A2, F4/A1, C3/A2, C4/A1, P3/A2, P4/A1, O1/A2, O2/A1, LOC/A2 and ROC/A1), and two electromyogram electrodes were attached under the chin to measure muscle tone. PSG data were digitized at 256 Hz and visually scored in 30-s epochs according to the sleep staging criteria of Rechtschaffen and Kales^82^. Sleep architecture variables included minutes and percentage of Stage 1, Stage 2, slow wave sleep (SWS) and rapid eye movement (REM), as well as total sleep time (TST), sleep latency (SL), and sleep efficiency (SE). Participants were excluded if they did not fall asleep during their first nap (*n* = 2), or if 2 out of 3 naps had TST less than 20 min and SE less than 35% (*n* = 1).

EEG data were preprocessed and analyzed using BrainVision Analyzer 2.0 (BrainProducts, Munich Germany) and Matlab 2011b (MathWorks, Natick MA). EEG data were bandpass filtered between 0.3 and 35 Hz, and a 60 Hz notch filter was also used to eliminate potential background noise. All epochs with artifacts and arousals were identified by visual inspection and rejected. Sleep spindles were automatically detected during Stage 2 and SWS using a wavelet-based algorithm developed by Wamsley and colleagues^57^. In short, the EEG signal underwent a time-frequency transformation using an 8-parameter complex Morlet wavelet. The wavelet scale corresponding approximately to the 10–16 Hz frequency range was extracted and used for spindle detection using Wamsley *et al*.’s^57^ thresholding algorithm. Following spindle detection, spindle densities were calculated by dividing the number of discrete spindle events by minutes spent in each sleep stage at each scalp EEG electrode site. Data for an individual channel were excluded if the channel was determined to be unreliable (e.g., became detached during the recording) based on visual inspection.

Power spectral density (μV^2^/Hz) was calculated by Fast Fourier Transform (FFT), applying a Hanning window to successive 3 sec epochs of sleep with 50% overlap. Spectral power was obtained for the following frequency bands: 0.5–1 Hz (slow oscillations; SO), 1–4 Hz (delta), 4–8 Hz (theta), 8–12 Hz (alpha), 12–15 Hz (sigma), and beta (15–30 Hz) during Stage 2, SWS, NREM (S2 + SWS combined) and REM sleep.

### Texture Discrimination Task (TDT)

Participants performed a texture discrimination task (TDT) similar to that developed by Karni and Sagi^38^. Visual stimuli for the TDT were created using the Psychophysics Toolbox^83,84^. Each stimulus contained two targets: a central letter (‘T’ or ‘L’), and a peripheral line array (vertical or horizontal orientation) in one of four quadrants (lower left, lower right, upper left, or upper right) at 2.5°−5.9° eccentricity from the center of the screen. The quadrant was counterbalanced across participants and visits. The peripheral array consisted of three diagonal bars that were either arranged in a horizontal or vertical array against a background of horizontally oriented background distracters, which created a texture difference between the target and the background.

An experimental *trial* consisted of the following sequence of four screens: central fixation cross, target screen for 33 ms, blank screen for a duration between 0 and 600 ms (the inter-stimulus-interval, or ISI), mask for 17 ms, followed by the response time interval (2,000 ms) and feedback (250 ms, red fixation cross with auditory beep for incorrect trials and green fixation cross for correct trials) before the next trial. Participants discriminated two targets per trial by reporting both the letter at central fixation (‘T’ or ‘L’) and the orientation of the peripheral array of three diagonal lines (horizontal or vertical) by making two key presses. The central task controlled for eye movements.

Each *block* consisted of 25 trials, each with the same ISI. A threshold was determined from the performance across 13 blocks, with a progressively shorter ISI, starting with 600 ms and ending with 0 ms. The specific sequence of ISIs across an entire session was [600, 500, 400, 300, 250, 200, 167, 150, 133, 100, 67, 33, 0]. A psychometric function of percent correct for each block was fit with a Weibull function to determine the ISI at which performance yielded 80% accuracy. TDT performance was calculated as the difference in threshold between Session 1 and Session 2, such that a positive score indicates performance improvement (i.e., decreased threshold in Session 2), whereas a negative score indicates deterioration^43,62^.

Participants were given task instructions and practiced the task during an orientation appointment prior to starting the study. During this practice, the peripheral target was located in a quadrant that would not be used during the study. This practice ensured that participants understood the task and aimed to reduce visit order effects due to general task learning that typically occurs the first time a participant performs a task. Additionally, on each study day, participants were allowed to practice an easy version of the task (ISI of 1,000-600 ms) to make sure they were able to discriminate the peripheral target between 90% and 100% correct before starting the actual task.

### Descending Subtraction Task (DST)

This task measures cognitive functioning for a brief (3 min) period of time by placing a considerable load on working memory while probing mental computation skills^29,30^. To begin, the experimenter gave the participant a three-digit number, for example “865”, which was repeated out loud by the participant. Then the participant was instructed to mentally subtract the number 9 from 865 and to say the answer (856) out loud. This number became the new minuend from which the subtrahend was subtracted. The subtrahend progressively decreased by 1 until it reached a value of 2, after which it returned to 9. Thus, on the next trial the participant should have subtracted the number 8 from 856. Participants were given 3 minutes to complete as many trials as possible. Instructions prompted the participant to work as fast and accurately as possible. The task was administered out loud with the experimenter writing the participant’s responses on a piece of paper attached to a clipboard so that the written responses were not visible to the participant. Participants were allowed to correct any response and were instructed to guess if they asked the experimenter for help. Total number of correct responses and number of correct responses as a proportion of total number of responses were calculated as indices of speed and accuracy, respectively. Difference scores (e.g., 5 min post-nap minus pre-nap performance) were calculated and reported as Δaccuracy and Δspeed.

### Data Reduction and Statistical Analyses

Participants whose first TDT threshold (i.e., Visit 1, Session1) was more than 2.5 standard deviations from the mean were flagged as outliers (*n* = 2 for main experiment and *n* = 2 for the Wake group; all were poor performers 2.5 standard deviations below the mean). In the main experiment, the two performance outliers together with the two participants removed for poor sleep and caffeine withdrawal were the bottom four TDT performers in the sample. Therefore, we employed equitable trim procedures and also removed the top four Visit 1, Session 1 performers (who were at ceiling). This left 48 participants whose Visit 1 data were analyzed (nap+ *n* = 26; nap− *n* = 22). We also applied equitable trim to the Wake group and removed the two poor-performing outliers as well as the top two performers, leaving 21 participants in the Wake group. One participant in the nap− group was excluded from DST analyses due to not understanding the task the first time it was performed (Visit 1 training), resulting in an accuracy difference score that was greater than 3 standard deviations above the mean.

Eight of the 48 participants in the nap intervention experiment were dropped or withdrew before study completion [due to illness (*n* = 1), changes in schedules that conflicted with the study (*n* = 3), noncompliance with the sleep-wake schedule (*n* = 2), and unknown reasons (*n* = 2)], leaving 40 participants who completed all three visits. Of the participants who completed the experiment, 21 were assigned to the Practice group (11 nap+, 10 nap−) and 19 were assigned to the Restriction group (9 nap+, 10 nap−). Demographics and other characteristics of our final samples are reported in Supplementary Table 1.

For spindle results, we focused on centro-parietal regions where spindles (in particular fast spindles) are known to be maximal^85–87^ and averaged the number of spindles detected over central and parietal electrodes within each hemisphere (i.e., C3-P3_avg_ and C4-P4_avg_). If no hemispheric differences were evident, we used the grand average (C3-C4-P3-P4_avg_). For power spectral analysis, due to significant topographic differences in power between the frontal and central regions (but no hemispheric differences), we averaged frontal (F3-F4_avg_) and central (C3-C4_avg_) electrodes.

Differences between nap+ and nap− at Visit 1 were tested using independent samples *t*-tests. Change across visits was tested using mixed-model ANOVA with Visit as a repeated measure and two between-participants factors: Nap Frequency group (nap+/−) and Intervention group (Practice/Restriction). Magnitude of performance change on the TDT was compared to zero (i.e., no change) using one-sample *t*-tests with difference scores. Bivariate Pearson correlations examined associations between TDT performance and nap sleep features at Visit 1. In order to reduce the number of correlations tested, we focused on power spectra during NREM (Stage 2 + SWS combined) and REM sleep stages, and chose specific frequency bands of interest for each sleep stage (NREM: SO, delta, and sigma and REM: theta). To test for moderation, we ran correlations for nap+ and nap− groups separately, then tested for significant differences in correlation coefficients using the Fisher *r*-to-*z* transform and *z*-test^88^. For analyzing spindles and power spectra across three visits, we used mixed-model ANOVA (see above) and specifically only tested variables that showed significant differences and/or moderation during Visit 1.

Due to the longitudinal nature of this study, there are missing data for sessions and visits, including bad electrodes during nap recordings, missing KSS scores and DST data due to experimenter error, and missing actigraphy data due to watch malfunction. Additionally, not all participants had every stage of sleep in their nap (no SWS: Visit 1 *n* = 3, Visit 2 *n* = 4, Visit 3 *n* = 4; no REM: Visit 1 *n* = 10, Visit 2 *n* = 4, Visit 3 *n* = 5). As such, degrees of freedom vary across analyses.

## Electronic supplementary material

Supplementary Tables

Dataset 1

Dataset 2

## Data Availability

The data analyzed in this study are included as Supplementary Information. All other study materials are available upon request to the authors.
