# Supplementary material for: The impact of frequent napping and nap practice on sleep-dependent memory in humans
Source: Sci Rep. 2018 Oct 10;8:15053. doi: 10.1038/s41598-018-33209-0 (PMC6180010; doi:10.1038/s41598-018-33209-0)
Supplement: Supplementary file 1 — Supplementary Tables [file 41598_2018_33209_MOESM1_ESM.pdf]

Supplementary Information

The impact of frequent napping and nap practice on sleep-dependent memory in humans

Elizabeth A. McDevitt[1,2], Negin Sattari[1,3], Katherine A. Duggan[1,4], Nicola Cellini[5], Lauren N. Whitehurst[1], Chalani Perera[1], Nicholas Reihanabad[1], Samantha Granados[1], Lexus Hernandez[1,3], Sara C. Mednick[1,3]

[1] Department of Psychology, University of California, Riverside
Riverside, CA 92521

[2] Princeton Neuroscience Institute, Princeton University
Princeton, NJ 08544

[3] Department of Cognitive Sciences, University of California, Irvine
Irvine, CA 92697

[4] Department of Psychiatry, University of Pittsburgh School of Medicine
Pittsburgh, PA 15261

[5] Department of General Psychology, University of Padova
Via Venezia 8, 315131
Padova, Italy

SUPPLEMENTARY TABLES

Supplementary Table 1. Sample characteristics.

	**Visit 1 Baseline**	**Nap Practice**	**Nap Restriction**
**NAP+**	N=26 (16 F)<br>Age=21.0 ± 2.4 yrs<br>ESS=5.9 ± 1.9<br>rMEQ=14.7 ± 3.6	N=11 (7 F)<br>Age=20.8 ± 2.3 yrs<br>ESS=6.2 ± 2.1<br>rMEQ=14.5 ± 3.6	N=9 (6 F)<br>Age=20.9 ± 2.3 yrs<br>ESS=6.0 ± 2.3<br>rMEQ=15.2 ± 3.7
**NAP-**	N=22 (13 F)<br>Age=22.4 ± 3.7 yrs<br>ESS=6.1 ± 1.8<br>rMEQ=14.1 ± 3.6	N=10 (7 F)<br>Age=22.9 ± 3.7 yrs<br>ESS=5.9 ± 2.2<br>rMEQ=14.1 ± 3.5	N=10 (5 F)<br>Age=22.6 ± 3.8 yrs<br>ESS=6.3 ± 1.7<br>rMEQ=14.2 ± 3.6
**Wake**	N=21 (13 F)<br>Age=19.1 ± 1.2 yrs<br>ESS=6.6 ± 2.5<br>rMEQ=12.7 ± 3.1	--	--

Visit 1 baseline data are for all participants included in the Visit 1 baseline analyses; Nap Practice and Restriction columns are the participants who completed all three visits. ESS = Epworth Sleepiness Scale; rMEQ = reduced Morningness-Eveningness Questionnaire. Values (except *N*) are *M* ± *SD.*

Supplementary Table 2. Visit 1 nap polysomnography sleep variables.

	NAP+	NAP-	Statistic
TST (min)	82.9 (18.6)	82.3 (17.9)	t_{46}=-0.11, p=.91
Stage 1 (min)	8.0 (5.0)	7.2 (4.1)	t_{46}=-0.58, p=.57
Stage 2 (min)	41.1 (14.4)	37.5 (12.0)	t_{46}=-0.91, p=.37
SWS (min)	20.4 (10.9)	24.8 (3.7)	t_{46}=1.04, p=.31
REM (min)	13.5 (9.8)	12.8 (2.4)	t_{46}=-0.24, p=.82
SL (min)	7.4 (8.9)	5.3 (0.87)	t_{46}=-1.03, p=.31
WASO (min)	12.9 (15.3)	13.5 (3.9)	t_{46}=0.12, p=.91
SE (%)	80.2 (19.1)	81.7 (4.0)	t_{46}=0.28, p=.78

TST = total sleep time; SWS = slow wave sleep; REM = rapid eye movement; SL = sleep latency; WASO = wake after sleep onset; SE = sleep efficiency. Values are *M* (*SD*).

Supplementary Table 3. Visit 1 prior night's sleep actigraphy variables.

	NAP+	NAP-	Statistic
TST (min)	370.4 (50.6)	389.7 (47.2)	t_{41}=1.28, p=.21
SL (min)	13.1 (12.3)	8.1 (12.3)	t_{41}=-1.33, p=.19
WASO (min)	57.2 (30.2)	61.2 (30.9)	t_{41}=0.43, p=.67
SE (%)	84.1 (7.2)	85.0 (6.8)	t_{41}=0.43, p=.67

These are data from one night prior to Visit 1. There were no differences between groups for any actigraphy variable. TST = total sleep time; SL = sleep latency; WASO = wake after sleep onset; SE = sleep efficiency. Values are *M* (*SD*).

Supplementary Table 4. Weekly nighttime sleep in each group across the five-week study.

	Practice					Restriction				
	BL	W1	W2	W3	W4	BL	W1	W2	W3	W4
**NAP+**										
TST (min)	386.0 (32.9)	379.9 (48.3)	376.6 (36.8)	375.3 (48.4)	388.4 (44.1)	362.3 (30.2)	357.8 (47.4)	387.9 (42.6)	379.7 (52.6)	379.0 (27.5)
SL (min)	9.1 (6.2)	14.6 (7.3)	9.0 (5.4)	12.6 (12.7)	10.6 (5.6)	15.7 (8.2)	14.0 (9.6)	10.9 (7.4)	14.1 (15.4)	18.8 (14.3)
WASO (min)	56.7 (18.7)	53.5 (11.1)	51.1 (11.9)	51.0 (19.6)	52.4 (13.0)	81.4 (30.0)	89.4 (47.8)	80.4 (15.5)	79.5 (23.7)	78.4 (19.2)
SE (%)	85.6 (4.8)	84.7 (2.8)	86.5 (2.4)	84.7 (6.5)	85.9 (3.1)	78.9 (6.4)	77.8 (10.4)	80.8 (3.4)	80.1 (6.6)	79.3 (4.9)
**NAP-**										
TST (min)	410.2 (37.1)	396.3 (60.7)	398.6 (34.1)	386.2 (45.7)	393.7 (52.7)	406.7 (32.5)	399.1 (44.4)	395.7 (46.4)	382.8 (39.2)	388.7 (39.9)
SL (min)	10.1 (7.5)	8.0 (8.1)	6.8 (5.0)	10.4 (8.8)	11.6 (10.1)	11.0 (7.2)	9.6 (3.9)	12.5 (6.8)	12.3 (13.7)	10.6 (6.2)
WASO (min)	56.0 (11.5)	61.9 (15.2)	59.6 (17.0)	58.2 (17.0)	62.8 (17.2)	65.2 (23.6)	63.9 (27.7)	60.7 (17.3)	63.3 (23.1)	67.3 (18.6)
SE (%)	86.2 (2.1)	84.7 (3.0)	85.9 (3.2)	84.7 (3.4)	84.2 (3.4)	84.3 (5.2)	84.3 (5.7)	84.4 (4.6)	83.4 (5.2)	83.3 (5.0)

These data were computed by first averaging nightly actigraphy data by week (BL, W1, W2, etc.) within each participant, then averaging those values across participants within the four groups. Only data from participants who completed all three visits are included in this table. The *N* included in each cell varies and may not represent the same subset of participants since actigraphs frequently malfunctioned. For example, participant 5 might be missing data for W1, and participant 8 is missing data for W3. For this reason, we did not calculate inferential statistics for these data; this table is meant to be purely descriptive. TST = total sleep time; SL = sleep latency; WASO = wake after sleep onset; SE = sleep efficiency. Values are *M* (*SD*).

Supplementary Table 5. Nap polysomnography sleep variables across three visits.

	Visit 1		Visit 2		Visit 3	
	Practice	Restriction	Practice	Restriction	Practice	Restriction
**NAP+**						
TST (min)	87.2 (16.9)	86.5 (9.1)	91.6 (11.7)	78.4 (26.6)	91.0 (16.0)	93.6 (4.9)
Stage 1 (min)	6.4 (4.0)	10.6 (5.5)	6.3 (4.2)	10.3 (7.7)	5.6 (4.2)	11.5 (6.2)
Stage 2 (min)	40.4 (12.2)	45.4 (9.1)	38.0 (14.3)	41.9 (14.0)	37.1 (17.8)	45.3 (17.2)
SWS (min)	23.3 (11.7)	16.6 (9.6)	26.0 (12.9)	11.7 (10.4)	28.7 (16.6)	21.8 (17.7)
REM (min)	17.1 (6.8)	13.9 (12.1)	21.2 (14.3)	14.6 (8.4)	19.5 (12.0)	15.0 (8.9)
SL (min)	4.4 (4.2)	7.3 (4.8)	5.3 (9.2)	2.6 (2.1)	1.4 (1.4)	3.2 (2.4)
WASO (min)	8.1 (17.2)	15.5 (13.1)	4.0 (4.9)	12.8 (14.5)	6.8 (11.0)	7.2 (5.4)
SE (%)	87.3 (18.0)	79.3 (12.3)	90.8 (11.0)	80.4 (25.7)	91.3 (13.4)	90.2 (5.6)
**NAP-**						
TST (min)	83.0 (20.1)	80.4 (18.1)	92.6 (10.5)	82.7 (22.8)	83.4 (26.9)	84.2 (19.7)
Stage 1 (min)	6.8 (3.4)	7.9 (4.9)	6.8 (3.4)	4.9 (2.5)	7.0 (4.0)	7.6 (3.4)
Stage 2 (min)	36.8 (13.8)	37.4 (11.3)	41.6 (14.0)	44.8 (15.1)	42.8 (17.1)	44.2 (17.8)
SWS (min)	28.8 (17.5)	20.2 (17.9)	26.8 (19.7)	19.7 (18.6)	17.7 (13.3)	21.7 (18.4)
REM (min)	10.6 (9.3)	14.9 (13.7)	17.4 (9.6)	13.4 (12.5)	16.0 (9.9)	10.8 (9.0)
SL (min)	4.0 (3.0)	6.7 (5.0)	5.2 (4.7)	4.7 (2.3)	4.2 (7.2)	7.8 (9.2)
WASO (min)	12.0 (17.5)	16.3 (21.4)	5.6 (6.8)	10.8 (10.8)	11.3 (20.4)	10.5 (11.6)
SE (%)	83.6 (18.8)	78.5 (21.4)	89.7 (9.2)	82.6 (17.2)	84.4 (27.0)	81.9 (18.1)

Only data from participants who completed all three visits are included in this table. One nap+ participant in the Practice group did not have PSG data recorded during Visit 2 due to technical failure. TST = total sleep time; SWS = slow wave sleep; REM = rapid eye movement; SL = sleep latency; WASO = wake after sleep onset; SE = sleep efficiency. Values are *M* (*SD*).
